# Valorization of recycled concrete powder, clay brick powder, and volcanic pumice powder in sustainable geopolymer concrete

**DOI:** 10.1038/s41598-025-93598-x

**Published:** 2025-04-01

**Authors:** Ahmed M. Tahwia, Mohamed Abdellatief, Aml Salah, Osama Youssf

**Affiliations:** 1 Department of Structural Engineering, Faculty of Engineering, Mansoura University, Mansoura, 35516 Egypt; 2 Department of Civil Engineering, Higher Future Institute of Engineering and Technology in Mansoura, Mansoura, Egypt

**Keywords:** Geopolymer concrete, Fly ash, GGBS, Recycled concrete powder, Clay brick powder, Volcanic pumice powder, Durability, Fire resistance, Civil engineering, Environmental social sciences, Materials science

## Abstract

The use of recycled powder as a binder in geopolymer concrete (GPC) represents a promising approach to reducing construction waste and promoting the production of sustainable materials. This study examines the impact of recycled concrete powder (RCP), clay brick powder (CBP), and volcanic pumice powder (VPP) on the mechanical, durability, and thermal properties of GPC with fly ash and slag under water curing. Key mechanical properties, including compressive strength (CS), splitting tensile strength, and flexural strength, were evaluated, while durability was assessed through water absorption, water penetration, and resistance to sulfate and acid attacks. The thermal performance was tested by exposing the samples to elevated temperatures of 200 °C, 400 °C, and 600 °C. Results demonstrated that incorporating 25% RCP enhanced CS by 14.88% at 28 days compared to the control mixture, although higher replacement levels (50% and 75%) led to reduced CS due to increased porosity. Similarly, CBP at 25% substitution resulted in a 21.12% increase in CS, with declines observed at higher replacement levels. Conversely, VPP at 25% substitution decreased CS by 8.68% at 28 days, with further significant reductions at higher levels due to its high porosity. Sulfate resistance testing in a 5% MgSO₄ solution showed minimal mass loss for CBP mixtures (0.3–1.2%) and moderate CS reductions (5.7–29.6%). RCP mixtures exhibited low mass loss (0.3–1.7%) and CS reductions (8.7–15.8%), while VPP mixtures experienced the highest mass losses (1.36–3.4%) and CS reductions (20.5–31.3%). SEM analysis revealed that RCP and CBP mixtures exhibited denser microstructures, which contributed to their enhanced durability and thermal stability. Generally, optimizing the replacement levels of RCP, CBP, and VPP improves the durability, pore structure, and mechanical performance of GPC. Among the materials, CBP demonstrated superior resistance in acidic environments, while RCP excelled in thermal stability, demonstrating their potential for producing sustainable and durable geopolymer concrete.

## Introduction

Concrete is valued for its compressive strength (CS), durability, and low porosity, making it ideal for demanding structures like high-rise buildings and bridges. However, traditional concrete production raises environmental concerns due to its reliance on Portland cement (PC), a major source of carbon emissions and resource depletion^1–3^. Therefore, geopolymer concrete (GPC), which uses industrial by-products like fly ash (FA) and ground granulated blast furnace slag (GGBS), offers a sustainable alternative^4–9^. Replacing PC with these waste-derived binders enhances mechanical properties while reducing environmental impact and conserving natural resources. With increasing emphasis on sustainability, GPC presents a cost-effective and eco-friendly solution for the construction industry.

### Recycled concrete powder (RCP)

The production of PC generates significant CO_2_ emissions, negatively impacting the environment. RCP, a by-product of concrete demolition and recycling, offers a promising solution to reduce CO_2_ emissions and promote sustainability. As a major form of solid waste, RCP is often underutilized, with much of it either stockpiled or sent to landfills, incurring disposal costs of $50–$100 per ton^10^. Due to its fine particle size and high pozzolanic properties (from amorphous silica and alumina), RCP is an effective material for replacing part of the binder in both PC and GPC^10–12^. He et al.^13^ stated that incorporating 30% RCP resulted in only a 3.91% reduction in CS at 28 days, related to the high amorphous silica content of RCP. This suggests that RCP may contribute to the pozzolanic reaction (*second hydration*), which is supported by other studies. Specifically, RCP contains active oxides such as CaO and SiO₂, along with un-hydrated PC particles, which react to form new hydrated compounds, including calcium silicate hydrate (C–S–H) and carboaluminate phases. Additionally, the fine particles of RCP (*less than 10 μm*) contribute a filling effect that promotes PC hydration by providing nucleation sites, densifying the microstructure, and improving CS. However, the filling effect is more pronounced at early hydration stages, while larger particles with low reactivity have limited filling capacity, resulting in a more significant reduction in CS^10,11,13–15^. Research has also shown that the presence of calcium (Ca^2^⁺) compounds in raw materials enhances the mechanical properties of GPC, as the geopolymer gel interacts with C–S–H and calcium aluminate hydrate (C–A–H) gels, improving overall strength^16,17^. However, limited studies have explored RCP in geopolymerization. The studies by Wu et al.^18^and Liu et al.^19^demonstrate the potential of RCP as a sustainable binder in GPC, demonstrating its impact on microstructure, mechanical performance, and durability. Wu et al. found that replacing 25–50% of FA-GGBS with RCP or recycled paste powder enhanced ductility and ultimate stress/strain, while 100% replacement led to reduced polymerization and performance^18^.

### Clay brick powder (CBP)

CBP is another promising waste material that has gained attention for use in GPC. Like RCP, clay bricks are commonly discarded in landfills, contributing significantly to environmental issues. In fact, large quantities of brick waste are generated each year from construction and demolition activities, with most of it being landfilled or incinerated^11,20^. CBP has been identified as a potential partial replacement for traditional binders in GPC, owing to its high SiO_2_ and Al_2_O_3_ content, which contributes to its pozzolanic reactivity. When finely ground, CBP can react with alkali activators, forming a geopolymer binder that improves the mechanical properties and durability of GPC. Several studies have explored the use of CBP in GPC, demonstrating that its incorporation leads to a denser microstructure and enhanced CS^11,21^. For instance, Mahmoodi et al. optimized 100% red clay brick waste-based geopolymer binders using a novel mixture design targeting SiO₂/Al₂O₃, Na₂O/SiO₂, and liquid/solid ratios. Fresh and mechanical properties were evaluated under ambient and elevated curing (50–100 °C). Additions of fly ash, metakaolin, and GGBS enhanced geopolymerization, with optimal SiO₂/Al₂O₃ (7.1) and Na₂O/SiO₂ (0.24) ratios achieving high strength via N–A–S–H/C–A–S–H formation, especially at 75 °C curing. Additionally, Dang et al.^21^ and Elemam et al.^22^ also suggest that CBP, when used in combination with other waste materials such as FA or GGBS, can further enhance the performance of GPC while promoting sustainability.

### Volcanic pumice powder (VPP)

VPP is an emerging waste material that has attracted considerable attention for its potential use in GPC. Volcanic pumice, a lightweight, porous volcanic rock, is widely available and commonly discarded after extraction, contributing to environmental concerns when landfilled^23^. VPP is rich in SiO_2_ and Al_2_O_3_, which are key components that confer pozzolanic reactivity, making it a promising substitute for conventional binders in GPC. Previous studies^23–25^ observed that substituting VPP for part of the binder in GPC resulted in a denser microstructure, reducing porosity and enhancing strength. Similarly, Kizilkanat et al.^26^investigated the effects of VPP and FA as supplementary cementitious materials in cement mortars, focusing on fresh properties, strength development, drying shrinkage, sulfate resistance, and chloride ion permeability. Replacing 15%, 25%, and 35% of PC with VPP and FA revealed that VPP negatively impacted paste consistency due to its porous structure. However, VPP-enhanced mortars demonstrated superior strength development, sulfate resistance, reduced drying shrinkage, and lower chloride ion permeability compared to FA mortars. The high silica content of VPP, coupled with its ability to react with CH in the hydration process, contributes to the formation of additional C–S–H gels, which help reduce pore volume and improve durability. On the other hand, Karaaslan et al.^27,28^investigated the durability enhancement of VPP-based GPC by incorporating calcium aluminate cement (CAC) as a partial replacement (up to 20%) for VPP. Results showed that a 20% CAC substitution significantly increased 28-day compressive strength from 20.0 to 70.0 MPa. Additionally, CAC improved durability by forming C–A–S–H gels, reducing permeability, and mitigating strength losses from freeze–thaw (43.4% to 4.6%) and wetting–drying cycles (36.7% to 1.4%). The incorporation of VPP into GPC involves the formation of a geopolymer binder through reactions with alkaline activators, typically NaOH, that lead to the generation of alumino-silicate (primarily composed of C–A–S–H and N–A–S–H) gels^23^. The formation of these gels reduces the porosity and increases the density of the concrete matrix, leading to significant improvements in the CS and long-term durability.

As highlighted earlier, the gap in the current study lies in investigating the potential for replacing both FA and GGBS with high volumes of waste materials, specifically RCP, CBP, and VPP, in proportions ranging from 25 to 75%, for the production of GPC under water curing conditions. While previous studies have explored the individual use of these waste materials in GPC, limited research has been conducted on their combined effects as replacements for FA and GGBS in varying proportions. Moreover, the impact of high volumes of RCP, CBP, and VPP on both the fresh and hardened properties of GPC, such as workability, strength, durability, and fire resistance, remains insufficiently studied. Therefore, this research investigates the effects of these waste materials and their substitution rates on the microstructure and mechanical properties of GPC, with a particular focus on durability and fire resistance. The results of this study could significantly contribute to the enhanced value-added utilization of recycled waste powders in the production of sustainable GPC.

## Experimental program

### Materials

**Fig. 1:**
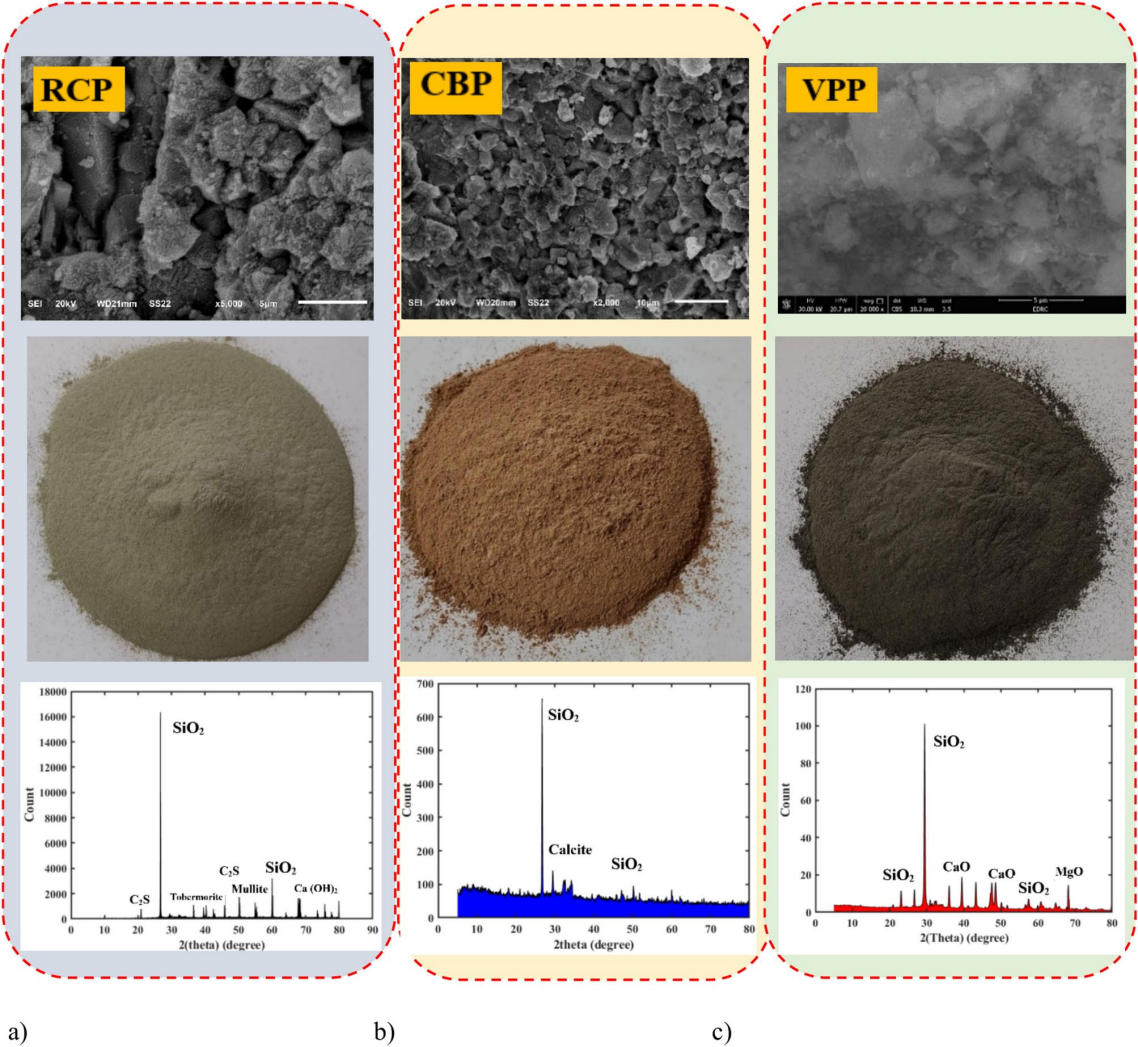
Micro, macro-structure and XRD patterns of solid waste materials: (**a**) RCP; (**b**) CBP, (**c**) VPP.

**Fig. 2:**
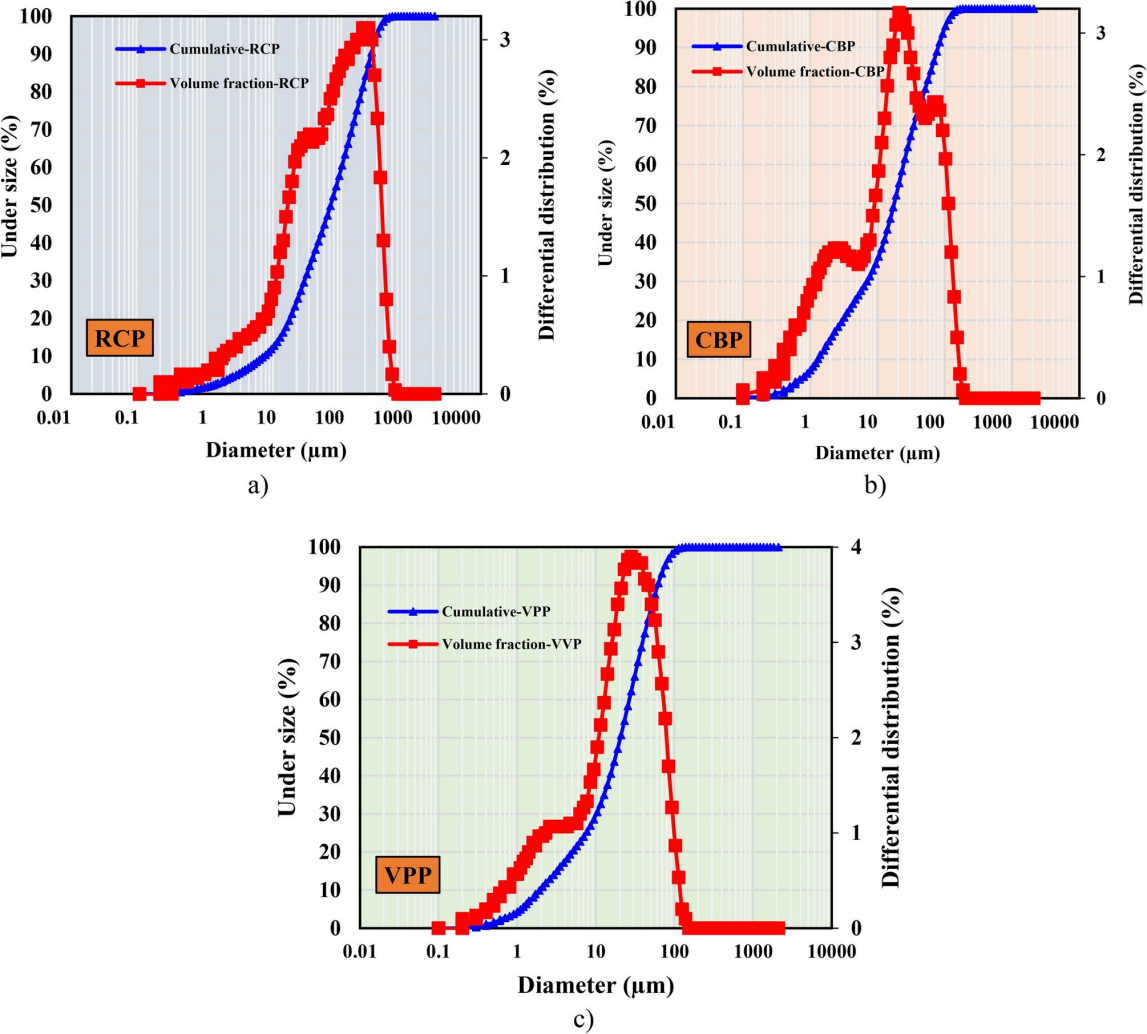
PSD of used waste materials.

The raw materials used in this investigation include GGBS, FA, silica fume (SF), RCP, CBP, VPP, reagent-grade 98% NaOH, aqueous sodium silicate (SS), superplasticizer (SP) and tap water. GGBS with a specific gravity of 2.9 and a surface area of 4960 cm^2^/g, FA with a specific gravity of 2.3 and a surface area of 5600 cm^2^/g, and SF with a specific gravity of 2.3 and a surface area of 200,000 cm^2^/g were used as precursors to produce GPC mixes in this study. RCP was obtained by collecting concrete waste from pre-manufactured concrete cubes, which were made of PC, fine aggregate, and coarse aggregate. The cubes were hand-broken, and the coarse aggregate was separated through filtration^42^. The remaining materials were then crushed in the lab until the desired fineness was achieved. Afterward, the crushed materials were filtered again to remove any impurities, resulting in the production of RCP (Fig. 1a). CBP was obtained by collecting building waste, which was then broken into smaller pieces. The pieces were ground in the lab and sifted to achieve the desired fineness, resulting in the production of CBP (Fig. 1b). VPP was sourced from Hurghada, Egypt, where pumice was broken into fragments, ground in the lab, and sifted to achieve the desired fineness (Fig. 1c). In the meantime, the surface morphology of these materials was examined using SEM, and the results are presented in Fig. 1. Additionally, the X-ray diffraction (XRD) patterns of RCP, CBP, and VPP revealed distinct mineralogical compositions that influence their effectiveness as binders in geopolymer concrete. RCP is characterized by crystalline phases such as calcium silicates and portlandite, reflecting its cementitious properties (Fig. 1a). CBP predominantly contains quartz and feldspar, which is indicative of its ceramic origin and lower reactivity than RCP (Fig. 1b). In contrast, VPP features an amorphous structure with minor crystalline phases, including feldspar and pyroxene, which demonstrate its high pozzolanic activity (Fig. 1c). Finally, the particle size distribution (PSD) of these raw materials is shown in Fig. 2. The average particle size (D_50_) of RCP, CBP, and VPP were 62.4 μm, 17.1 μm, and 20.9 μm, respectively. Table 1 presents the elemental composition of raw materials based on X-ray fluorescence (XRF) analysis. The major constituents of the RCP are SiO_2_ and CaO compounds, with smaller amounts of Al_2_O_3_ and Fe_2_O_3_ oxide, whereas the CBP and VPP consists mainly of SiO_2_ and Al_2_O_3_, with CaO and Fe_2_O_3_ oxide as minor components.

**Table 1:** Chemical composition of raw materials.

Element %	CaO	Al_2_O_3_	SiO_2_	MgO	Fe_2_O_3_	K_2_O	SO_3_	Na_2_O	TiO_2_	LOI
RCP	30.6	9.35	43.15	6.23	3.17	1.35	2.55	0.98	0.55	9.4
CBP	5.09	16.86	60.83	1.32	8.56	1.94	0.23	1.07	1.58	1.74
VPP	7.95	17.45	65.7	1.77	3.54	2.92	0.38	5.56	3.57	2.19
FA	4.33	24.56	54.32	1.23	3.85	1.33	0.15	0.98	1.53	3.85
SF	0.45	0.95	94.74	0.29	1.23	0.35	0.27	0.25	0.32	0.99
GGBS	39.98	13.65	31.74	5.94	0.36	0.48	1.53	0.88	0.62	1.82

Natural sand with a specific gravity of 2.6, fineness modulus of 2.73, and passing through sieve size of 4.75 mm was the fine aggregate in all mixes. A mixed solution including commercial Na_2_SiO_3_ (SS): Na_2_O = 15%, SiO_2_ = 37.5wt, and H_2_O = 47.5% with a SiO_2_/Na_2_O ratio of 2) with a specific gravity of 1.4, and NaOH was commercial grade with a purity level of (97–98%), with a specific gravity of 1.97, which was prepared with a molarity of 14 M. SP conforming to ASTM C494^35^ with a specific gravity of 1.07 was used as Type G to enhance the workability of the prepared mixes.

### GPC manufacturing and curing

**Fig. 3:**
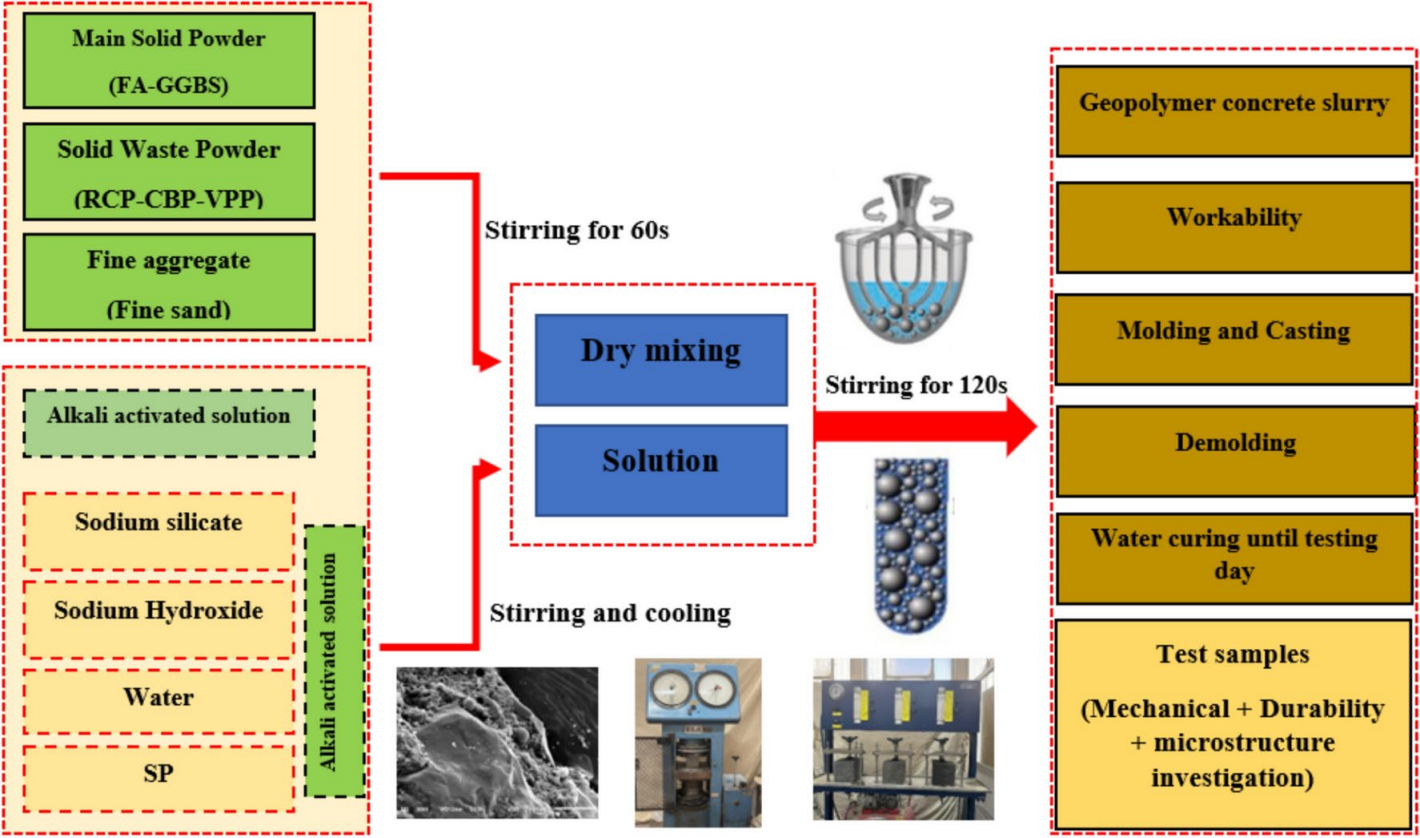
Mixing procedure of GPC mixtures.

This study explores the feasibility of replacing both FA and GGBS with high volumes (25–75%) of waste materials—RCP, CBP, and VPP—for producing GPC under water curing conditions. Ten mixtures were designed and prepared to achieve the objectives of the research and compare them with the reference mix, as presented in Fig. 3. Mixtures proportions of the prepared mixtures are presented in Table 2. The reference mix (M0) contained FA, GGBS, and SF. In the other mixes, the RCP, CBP, and VPP were incorporated, and implemented for partial replacement (25%, 50%, and 75%) of both FA and GGBS. In the mixing process according to ASTM C305^29^, a fixed 70-L horizontal mixer was used to mix the concrete components. The binder was put in the mixer and mixed for 1 min, then the sand was added and mixed for 1 min, then the alkaline solution was put in and mixed for 2 min, then SP was added. Finally, additional water was added to the mixer in an unspecified quantity until the desired slump value of 9.0 cm, consistent across all mixes, was achieved. After 24 h of casting, all samples were demolded and cured in water until the testing day.

**Table 2:** The quantity of GPC components (kg/m^3^).

Groups	MIX-ID	FA	GGBS	SF	RCP	CBP	VPP	Sand	NaOH	SS	SP
Control	M0	240	240	120	–	–	–	1405.3	38.4	171.43	9
G-I	RCP25	180	180		116.93	–	–				
	RCP50	120	120		233.88	–	–				
	RCP75	60	60		350.83	–	–				
G-II	CBP25	180	180		–	106.17	–				
	CBP50	120	120		–	212.36	–				
	CBP75	60	60		–	318.55	–				
G-III	VPP25	180	180		–	–	101.5				
	VPP50	120	120		–	–	203				
	VPP75	60	60		–	–	304.5				

### Test procedure

#### Fresh properties and bulk density

To maintain the desired workability with a slump value of 9.0 cm (*ASTM C143*), water demand is determined by gradually adding water to the mix while monitoring its workability. The slump test is conducted repeatedly during mixing by filling a standard cone mold with concrete, compacting it, and lifting the mold to measure the slump height. Water is adjusted until the target slump is achieved. According to *ASTM C138/C138M*, the bulk density test of GPC involves measuring the mass of mixed concrete in a standard container of known volume to determine its density.

##### Mechanical properties tests

**Fig. 4:**
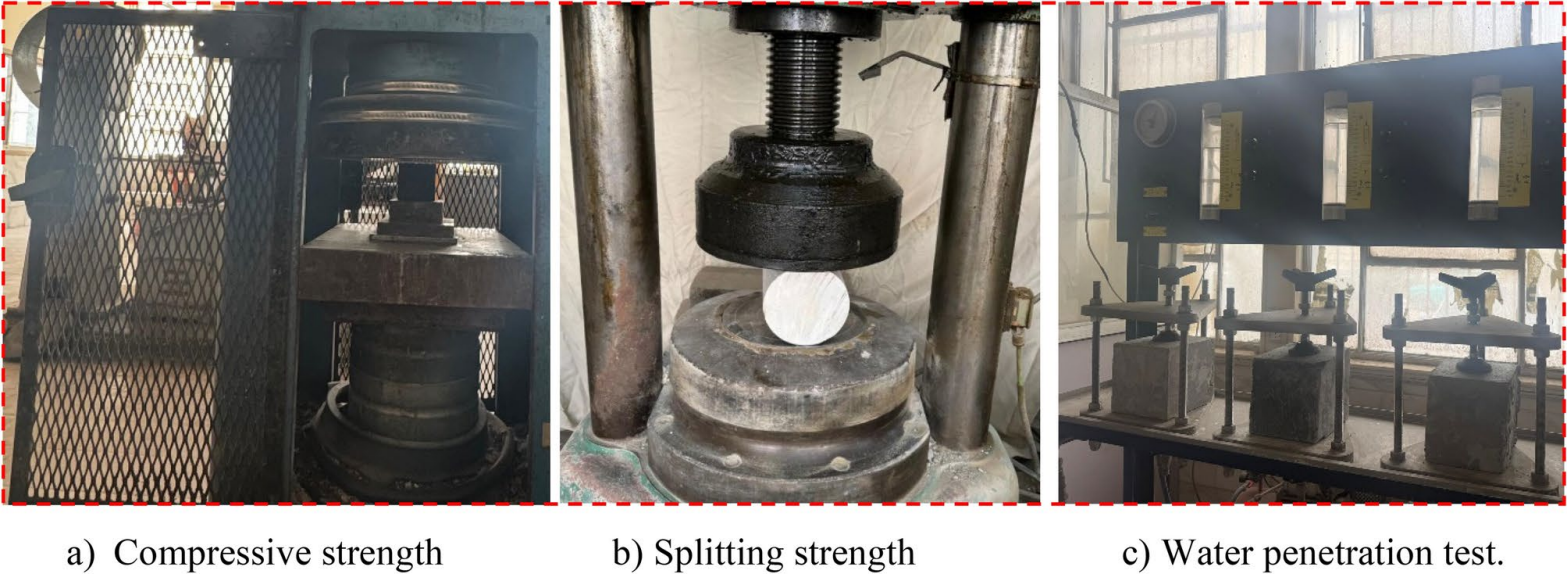
Experimental set up of GPC production.

The mechanical properties of the GPC were evaluated, including compressive strength, splitting tensile strength, and flexural strength. Compressive strength was tested at 7, 28, and 56 days using three 50 × 50 × 50 mm concrete cubes per mix (Fig. 4a), as per *ASTM C109/C109M*, with the average value recorded for each age. Splitting tensile strength was measured at 28 days using two 100 × 200 mm concrete cylinders per mix (Fig. 4b), following ASTM C496. Flexural strength was assessed at 28 days using three 40 × 40 × 160 mm concrete beams per mix, in accordance with ASTM C293.

##### Durability and elevated temperature resistance

The water absorption test was conducted according to *ASTM C642*, using three 50 mm cube samples per mix. Samples were oven-dried at 100 °C ± 5 °C for 24 h, then immersed in tap water for 24 h to determine their wet weight. Water absorption was calculated as the ratio of the absorbed water to the dry weight of the specimen. The water penetration test, based on *BS EN 12,390–8*, was performed to assess the concrete’s permeability. Three 150 mm cubes were tested after 28 days of curing. Water was applied under a pressure of 500 ± 50 kPa for 72 ± 2 h. After the test, the cubes were split, and the depth of water penetration was measured to evaluate permeability, as illustrated in Fig. 4c. For sulfate and acid attack tests, 50 × 50 × 50 mm cube samples were immersed in a 5% MgSO₄ solution for sulfate attack and a 5% H₂SO₄ solution for acid attack, following ASTM C267. The solutions were periodically stirred to maintain homogeneity throughout the 28-day testing period. After exposure, the samples were dried and weighed. The percentage losses in mass and compressive strength were calculated to evaluate the durability of all mixtures under these aggressive conditions.

**Fig. 5:**
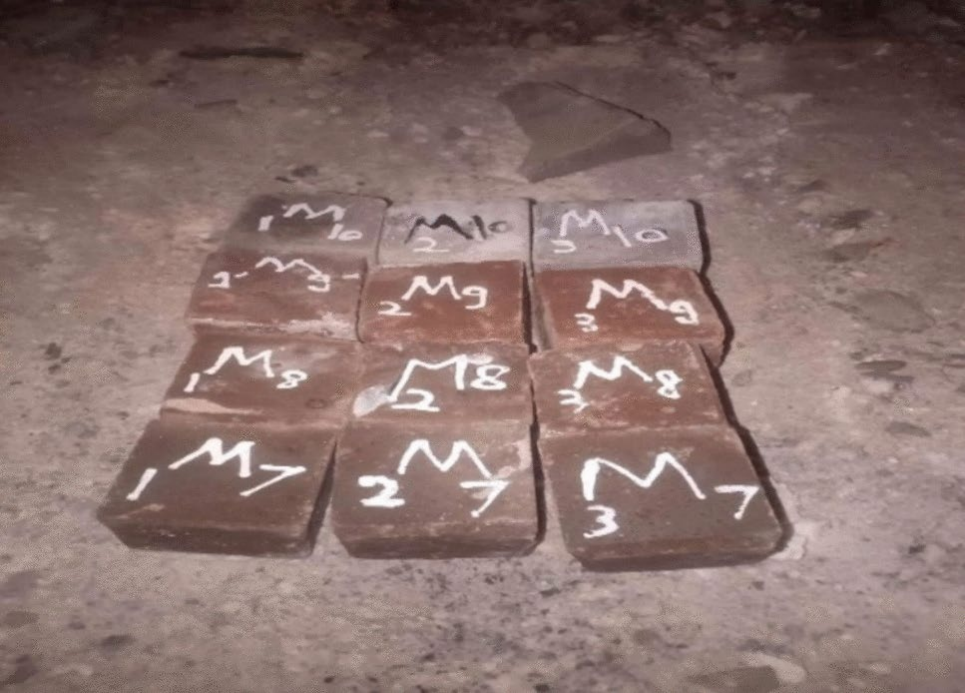
Experimental set up of GPC production.

Additionally, GPC samples were subjected to elevated temperatures to evaluate mass loss and residual compressive strength. After 28 days of curing, the samples were heated in a furnace with internal dimensions of 1100 × 800 × 600 mm. The furnace operated at a controlled heating rate of 15 °C per minute until the target temperatures of 200 °C, 400 °C, and 600 °C were reached. Samples were held at these temperatures for 1.5 h before the furnace door was opened to allow natural cooling (Fig. 5). After heating, the samples were weighed to calculate mass loss and left to cool at room temperature for 24 h prior to mechanical testing.

##### SEM morphology and XRD test

The morphology of small fragment specimens was examined using a *JEOL JSM-6510LV* scanning electron microscope with an accelerating voltage of 15.0 kV. It has a resolution of 3 nm, a magnification of 180–100,000 times, an acceleration voltage range of 0.5–30 kV and the working distance was adjusted between 9 and 15 mm for optimal imaging. A powder X-ray diffractometer (PANalytical X’Pert PRO) with CuK radiation (= 0.1541 nm) was used to examine crystalline phase development of samples. The X-ray (XRD) spectra were obtained using an operating voltage of 40 kV and a current of 40 mA with a 2theta range of 5° to 70°.

## Results and discussions

### Water demand and fresh density

**Fig. 6:**
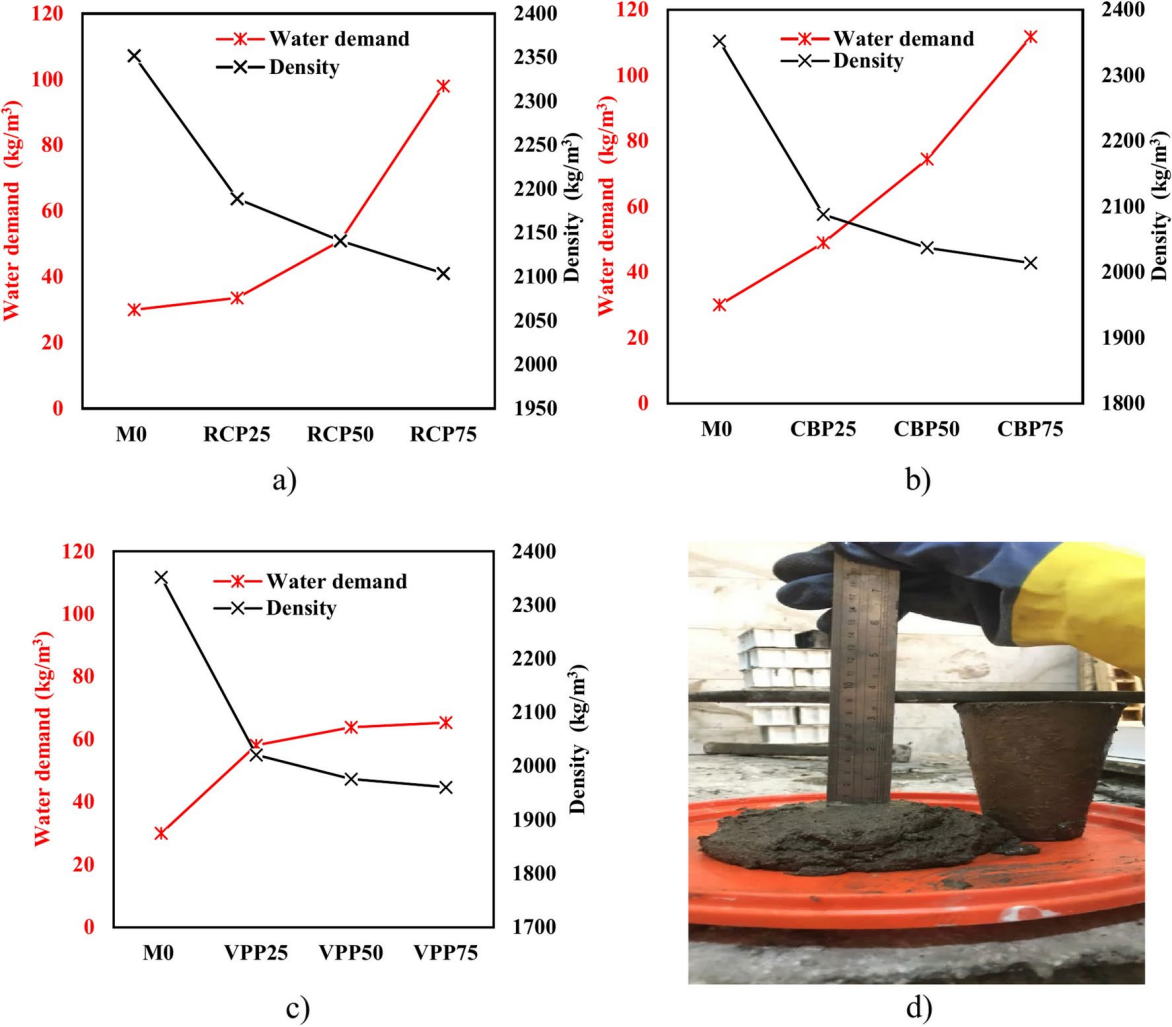
Effects of different waste materials on water demand and bulk density.

The variation in water demand for the different mixtures can be attributed to the distinct properties of RCP, CBP, and VPP, specifically their surface area, particle size distribution, and porosity^23^. The fine nature of RCP, with its high surface area, leads to increased water absorption, as the fine particles require more water for adequate workability. This is reflected in the water demand of the RCP75 sample (Fig. 6a), which increased from 30 to 97.96 kg/m^3^. Similarly, CBP (Fig. 6b), which is typically finer and more porous, exhibited the highest water demand (111.71 kg/m^3^). The presence of silica and alumina in CBP further contributes to this increase, as these materials interact with the alkaline solution in GPC, requiring more water to maintain workability^19,22^. In contrast, VPP (Fig. 6c), despite its higher porosity, required less water for workability (65.34 kg/m^3^ in the VPP75 sample). This contradicts the expectation that higher porosity should increase water demand. However, its lower specific surface area and coarser particles facilitated better fluidity, reducing overall water absorption^24–26^. This finding aligns with previous research by Karaaslan et al.^28^, where pumice-based geopolymer mortars exhibited reduced workability due to pumice’s inherent porous nature but improved when combined with FA and calcium aluminate cement (CAC). Their study found that FA, due to its spherical particle shape, improved flowability, while CAC prevented particle agglomeration, enhancing cohesion and workability. Compared to Karaaslan et al.^28^, the current study reveals a more significant increase in water demand for CBP and RCP, indicating that these powders absorb more water than VPP. This difference is primarily due to the higher reactivity and finer particle size of CBP and RCP, which interact strongly with the alkaline activator. As shown in Fig. 6d, achieving a target slump of 9.0 cm required additional water, particularly for mixtures with higher CBP and RCP content. Therefore, optimizing the replacement levels of these materials is crucial to balancing workability and mechanical performance.

Regarding bulk density (Fig. 6), the fine particles in RCP, CBP, and VPP can lead to a denser mix by filling voids between larger aggregates, thereby increasing the overall mass per unit volume. However, the effect on density depends on the proportion of these powders and their interaction with other mix components. In the current study, the densities for high volumes of RCP75, CBP75, and VPP75 reached 2104 kg/m^3^, 2014 kg/m^3^, and 1961 kg/m^3^, respectively. This variation is attributed to the specific gravity differences among RCP, CBP, and VPP particles^10,19,30^.

### Mechanical performance

#### Mechanical performance of group-I

**Fig. 7:**
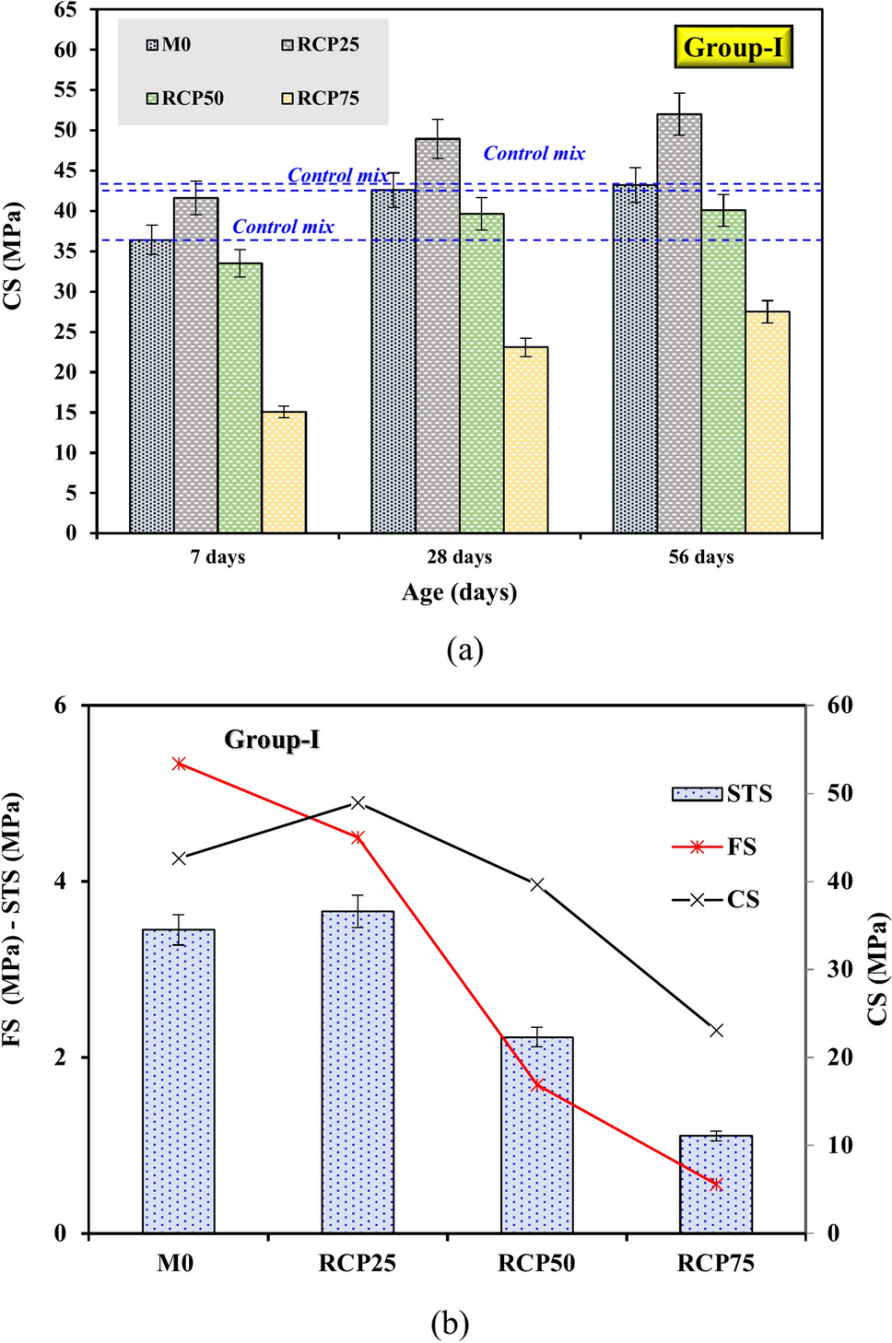
Effects of RCP substitution on (**a)** CS and (**b)** STS and FS at 28 days.

The compressive strength of GPC was measured at various curing ages, while the flexural and splitting tensile strengths were evaluated at 28 days. Figure 7a presents the CS results for samples tested at 7, 28, and 56 days. In the control mix (M0), the CS increased from 36.4 MPa at 7 days to 42.6 MPa at 28 days, an improvement of 17.8%. However, at 56 days, the strength marginally decreased by 1.4%, reaching 43.2 MPa. When 25% RCP was substituted for FA and GGBS in Group-I, the CS of the RCP25 sample improved significantly, showing an increase of 14.2% at 7 days and approximately 14.88% at 28 days compared to the M0. This improvement is attributed to RCP’s filler effect, where its fine particles enhance matrix densification by filling voids and reducing porosity^11,13^. Furthermore, RCP contains residual cementitious compounds, such as unhydrated cement and reactive silica, which contribute to secondary hydration reactions and strengthen the matrix. The optimized 25% replacement ensures a balance between maintaining sufficient cementitious material and utilizing the beneficial properties of RCP (Fig. 7a). Liu et al.^19^ demonstrated that while incorporating recycled powder reduces the CS of GPC, the use of these powders at optimized replacement levels (< 50%) can achieve acceptable mechanical performance. Consistently, the present investigation achieved a CS of 27.5 MPa using 75% RCP, aligning with^19^.

In terms of splitting tensile strengths (STS), the RCP25 sample demonstrated enhancements of 6.08% (4.5 MPa) at 28 days over the M0, while declining flexural strength (FS) by 0.84% (Fig. 7b). These improvements in the STS are similarly attributed to RCP’s filler effect and residual cementitious activity, which contribute to matrix densification and secondary hydration. However, as the RCP content increased to 50% and 75%, the strengths declined significantly. At 28 days, the CS dropped to 39.65 MPa (50% RCP) and 23.07 MPa (75% RCP), while FS and STSs also diminished. This reduction is due to the decreased cementitious content, as higher RCP levels replace reactive binders like FA and GGBS, leading to fewer hydration products, such as C–S–H and N–S–A–H, which are essential for strength development. Additionally, RCP particles often contain microcracks, impurities, and adhered old mortar, which weaken bond strength and contribute to a less cohesive matrix as discussed later. Excessive RCP also increases porosity, weakening the overall structure, as RCP’s limited pozzolanic reactivity cannot compensate for the reduction in primary binders at high substitution levels. The findings align with studies such as those by Kou and Poon^31^and Xiao et al.^32^, which observed that moderate RCP replacement enhances mechanical properties due to improved particle packing and secondary reactions, but excessive RCP adversely affects strength due to reduced binder reactivity and increased porosity.

##### Mechanical performance of group-II

**Fig. 8:**
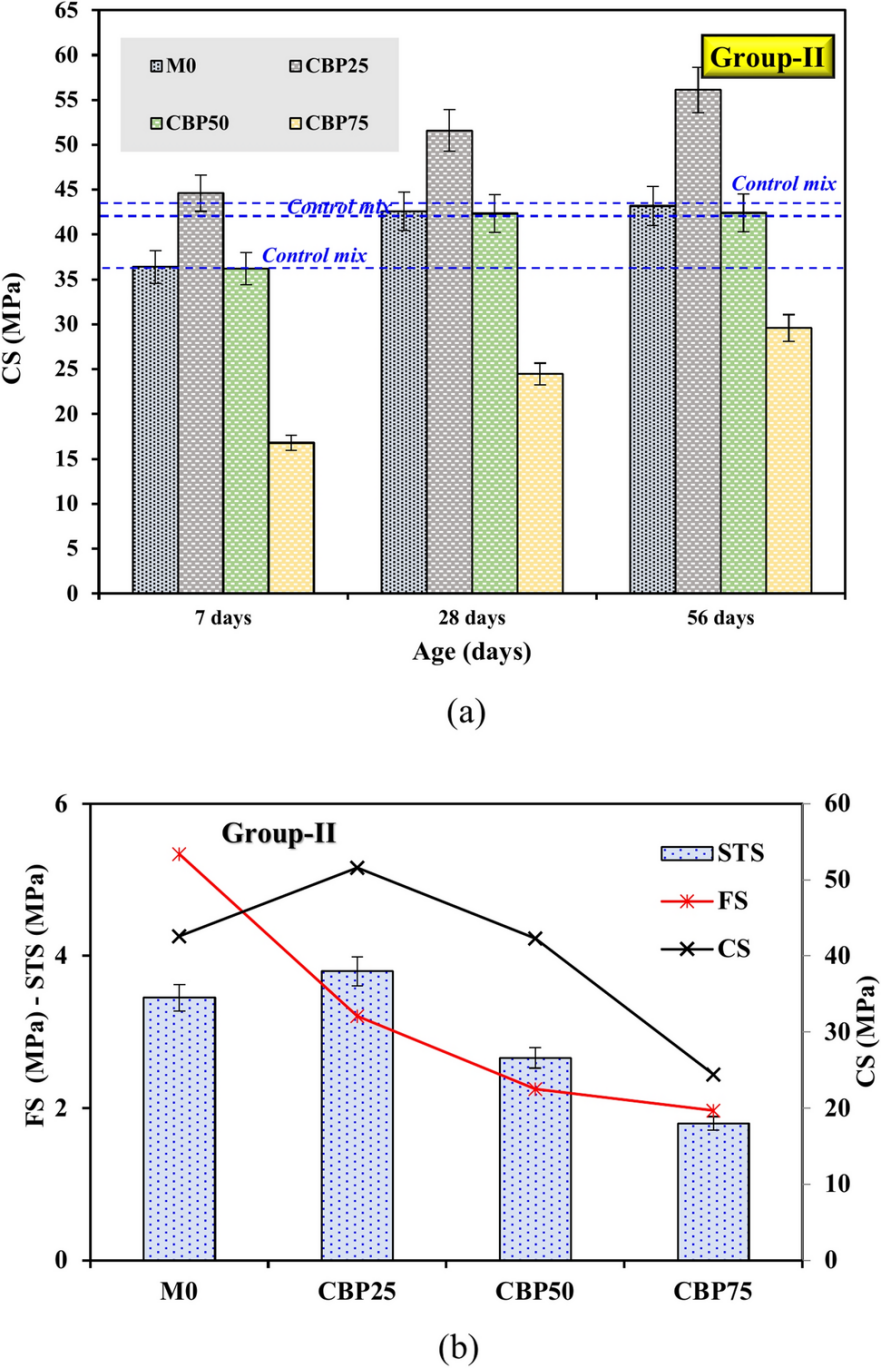
Effects of CBP substitution on (**a)** CS and (**b)** STS and FS at 28 days.

The CS, FS, and STSs of GPC were also analyzed for samples incorporating CBP at varying replacement levels, as shown in Fig. 8. When 25% CBP was substituted for FA and GGBS, the CS of the CBP25 sample improved by 22.5 (44.6 MPa) at 7 days and 21.12% (51.60 MPa) at 28 days compared to M0 (Fig. 8a). This improvement can be attributed to CBP’s pozzolanic reactivity and filler effect, where the fine particles enhance particle packing and matrix densification by filling voids and reducing porosity as recommend by^11,20,30,32^. Previous studies reported that the CBP particle contains reactive alumina and silica, which participate in secondary hydration reactions, contributing to strength development^30,32,33^.

In terms of STS, the CBP25 sample showed an increase of 10.14% at 28 days (3.8 MPa) compared to the M0, while the FS demonstrated a significant reduction of 4.13% (Fig. 8b). These enhancements of STS are primarily due to CBP’s role as a reactive filler that contributes to matrix densification and strength gain. However, as the CBP content increased to 50% and 75%, the strengths declined significantly. At 28 days, the CS dropped to 42.33 MPa (50% CBP) and 24.45 MPa (75% CBP), while FS and STS also showed noticeable reductions. This decrease is due to the reduced proportion of high-reactivity binders like FA and GGBS, resulting in fewer hydration products such as C–S–H, which are critical for strength. Additionally, CBP particles often contain impurities and are less reactive than primary binders, limiting their contribution to long-term strength. The increased porosity at higher CBP levels further compromises the mechanical properties, as CBP’s limited reactivity cannot fully compensate for the reduction in active binders. These findings align with studies by^11,19,32^, which observed that moderate CBP replacement enhances mechanical properties through improved particle packing and secondary hydration reactions, but excessive replacement leads to strength reductions due to lower binder reactivity and higher porosity. This demonstrates the importance of optimizing CBP content to achieve a balance between mechanical performance and sustainability in geopolymer concrete mixtures.

##### Mechanical performance of group-III

**Fig. 9:**
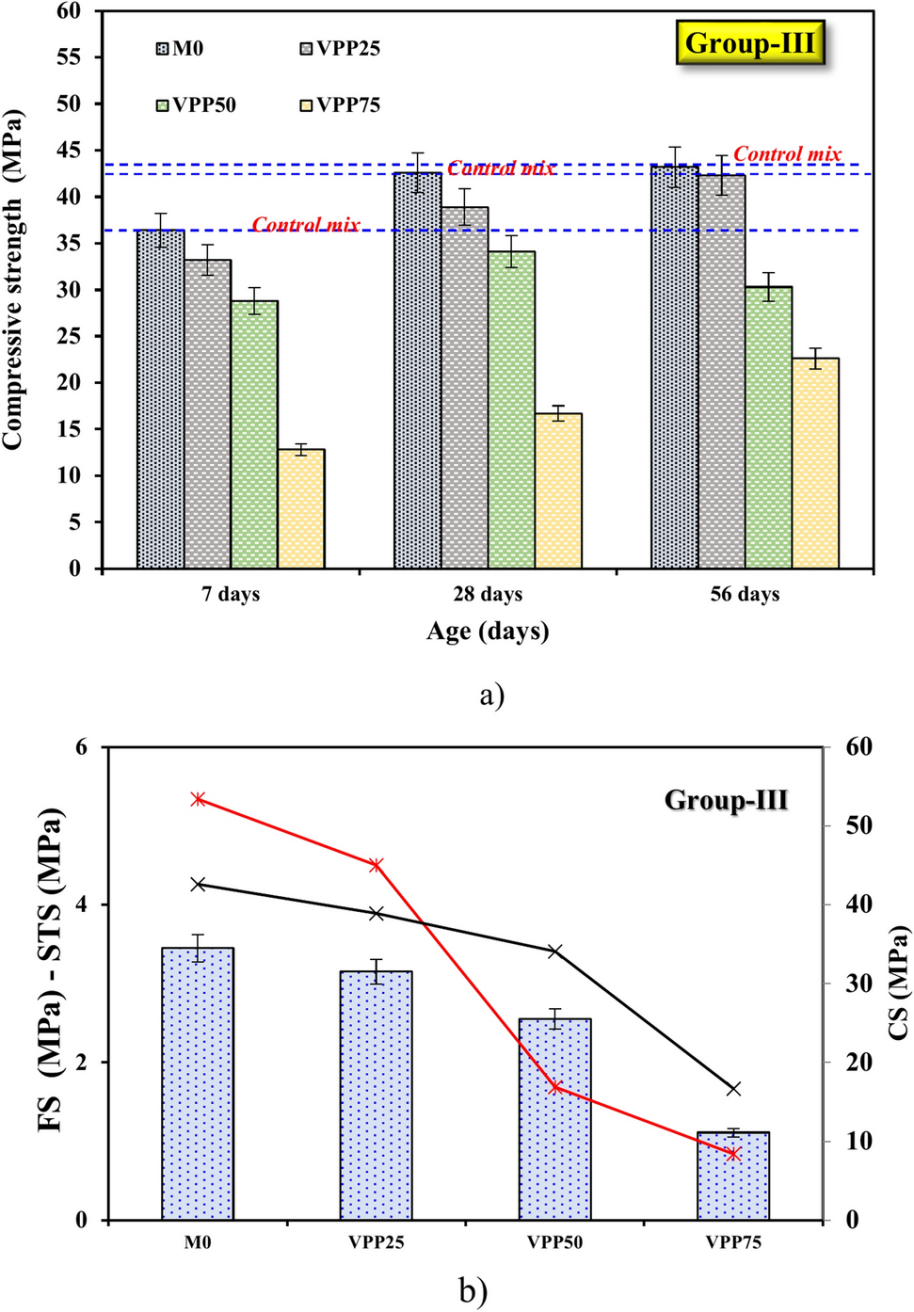
Effects of VPP substitution on (**a)** CS and (**b)** STS and FS at 28 days.

The CS, FS, and STSs of GPC were also analyzed for samples incorporating VPP at varying replacement levels, as shown in Fig. 9a. When 25% VPP was substituted for FA and GGBS, the CS of the VPP25 sample decreased by 8.79% at 7 days and 8.68% at 28 days compared to M0. This reduction can be attributed to the highly porous nature of pumice, which introduces voids into the matrix and reduces its overall density^24–26^. In terms of STS, the VPP25 sample exhibited a decline of 7.5% at 28 days (3.15 MPa) compared to M0, while the FS also decreased by 24.2%. These reductions in mechanical properties can be attributed to the weak interfacial bond strength of pumice particles, which contain inherent microcracks and a less reactive structure. The porous morphology of pumice (see Fig. 1), while potentially beneficial at low replacement levels, leads to an increase in voids within the matrix, compromising mechanical performance.

As the VPP content increased to 50% and 75%, the reductions in strength became more pronounced. At 28 days, the CS dropped to 34.1 MPa (50% VPP) and 16.69 MPa (75% VPP), while FS and STS showed significant declines Fig. 9b. Studies have demonstrated that incorporating VPP in concrete can enhance CS while potentially reducing STS under certain conditions. For instance, Zeyad et al.^34^ produced concrete with VPP, observing an increase in CS due to the filler effect and the reactive nature of pumice. The porous morphology of VPP enables effective interlocking within the matrix, enhancing particle packing and reducing porosity. This morphological advantage, coupled with the pozzolanic reactivity of VPP, contributes to refining the microstructure and increasing strength at earlier ages^34,35^. The reduction in CS at higher VPP contents, typically between 25 to 75%, can be attributed to the excessive porosity introduced by the pumice particles and the dilution of reactive binders like FA and GGBS. Similar findings were reported by^26,34,35^, where the limited pozzolanic activity of VPP at higher contents resulted in fewer hydration products, thus negatively affecting the overall strength development.

### Durability characteristic

#### Water absorption and penetration depth

**Fig. 10:**
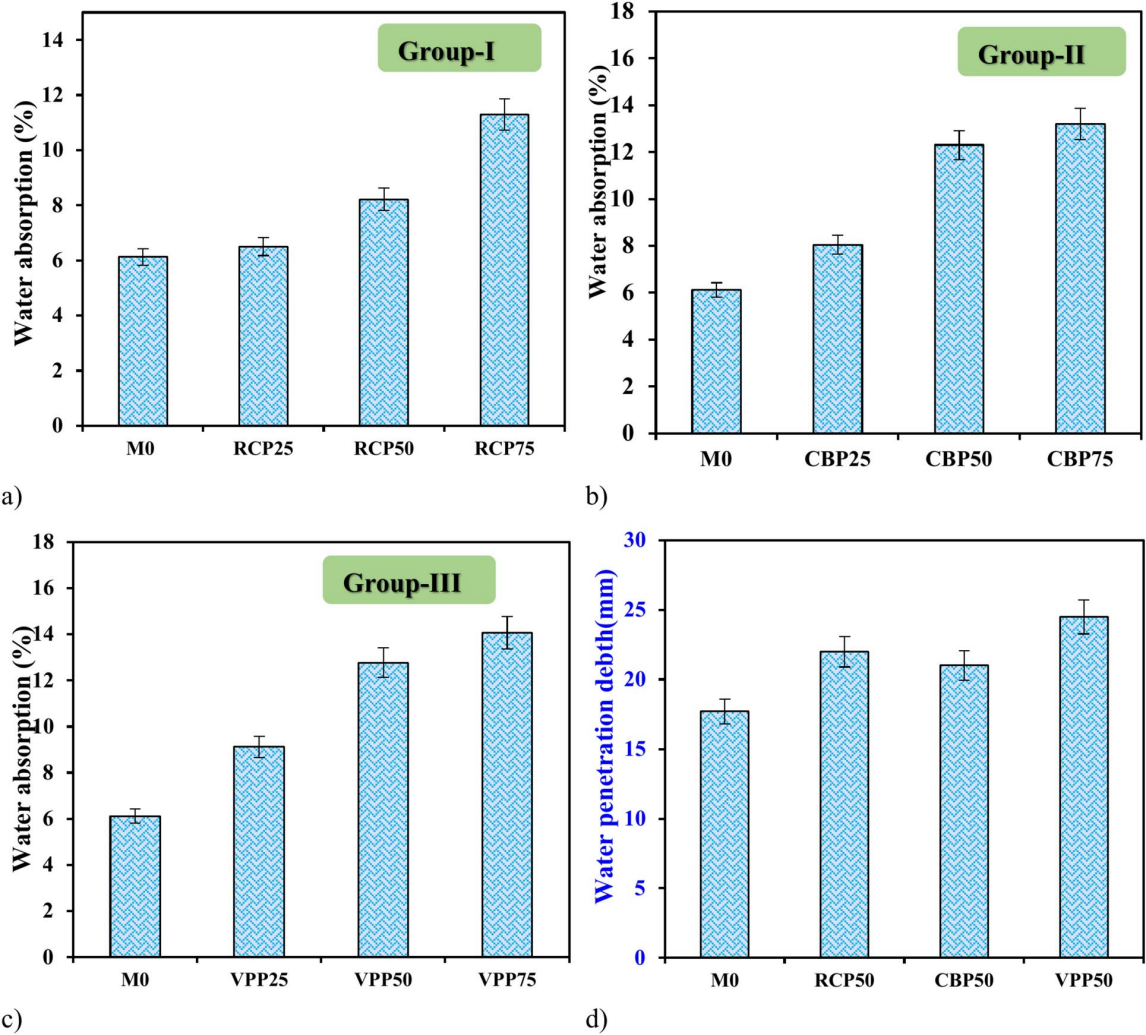
Water absorption of GPC containing (**a)** RCP, (**b)** CBP, and (**c)** VPP (**d)** Water penetration depth of GPC Containing 50% of waste materials.

Water absorption and penetration depth are critical indicators of concrete durability, particularly for protecting reinforcing bars in reinforced concrete structures. The results of this study, displayed in Fig. 10, reveal distinct effects of RCP, CBP, and VPP on GPC. The M0 exhibited a water absorption rate of 6.12% but incorporating RCP (RCP25– RCP75 samples) gradually increased water absorption reached 11.3%, attributed to RCP’s high porosity and residual adhered mortar, which enhance its water absorption capacity (Fig. 10a). This aligns with prior studies indicating that RCP particles, despite contributing to matrix densification, may increase permeability due to their porous structure. CBP (CBP25–CBP75 samples) incorporation caused the most significant rise in water absorption (Fig. 10b), reaching 13.2%, surpassing RCP, due to its inherently porous structure and micro-voids from fired clay, making it highly water-demanding^10–12,22,33^. In contrast, VPP (VPP25– VPP75 samples) displayed inconsistent behavior (Fig. 10c). Water absorption increased initially (VPP25) up to 9.12%, jumped significantly (VPP50) to 12.77%, and then rose slightly (VPP75) to 14.07%. This variability is likely due to VPP’s unique particle structure and pozzolanic activity, which can refine the pore structure and enhance matrix densification at specific replacement levels^34,35^. Previous studies support these observations, suggesting that volcanic materials can improve impermeability under optimized conditions but may exhibit variable performance depending on mix design^26,33,34^. Overall, VPP had the most adverse effect on water absorption and penetration depth, followed by CBP and RCP. These findings emphasize the need to optimize replacement ratios to balance durability and performance, consistent with earlier research on supplementary cementitious materials in geopolymer concrete.

The water penetration depth of GPC mixtures containing 50% waste materials reveals significant variations based on the type of material used. The control mixture exhibited a penetration depth of 17.7 mm, which increased with the incorporation of RCP, CBP, and VPP. For mixtures RCP50 sample, the penetration depth reached 22 mm, indicating a moderate increase. This can be attributed to the porous structure of RCP, which contains adhered mortar and microcracks that enhance water ingress. The CBP50 sample showed a slightly lower penetration depth of 21 mm compared to RCP. The micro voids inherent in fired clay brick particles make CBP highly porous and water-absorbent, which aligns with previous research demonstrating that CBP increases permeability and water absorption. However, CBP’s relatively smaller particle size and better compatibility with the cementitious matrix compared to RCP may contribute to a slightly reduced penetration depth^20,22^. The highest water penetration depth of 24.5 mm was observed in VPP50 sample (Fig. 10d). VPP’s lightweight and porous nature significantly increase water ingress, as supported by studies that demonstrate its high surface area and low density. Despite VPP’s pozzolanic activity, which can refine pore structure at optimal replacement levels, excessive amounts can lead to higher porosity and reduced resistance to water penetration. Previous has shown that volcanic materials may enhance impermeability when finely ground and used in smaller proportions but can have adverse effects when overused^34,35^.

#### Sulfate attack: weight and strength changes

**Fig. 11:**
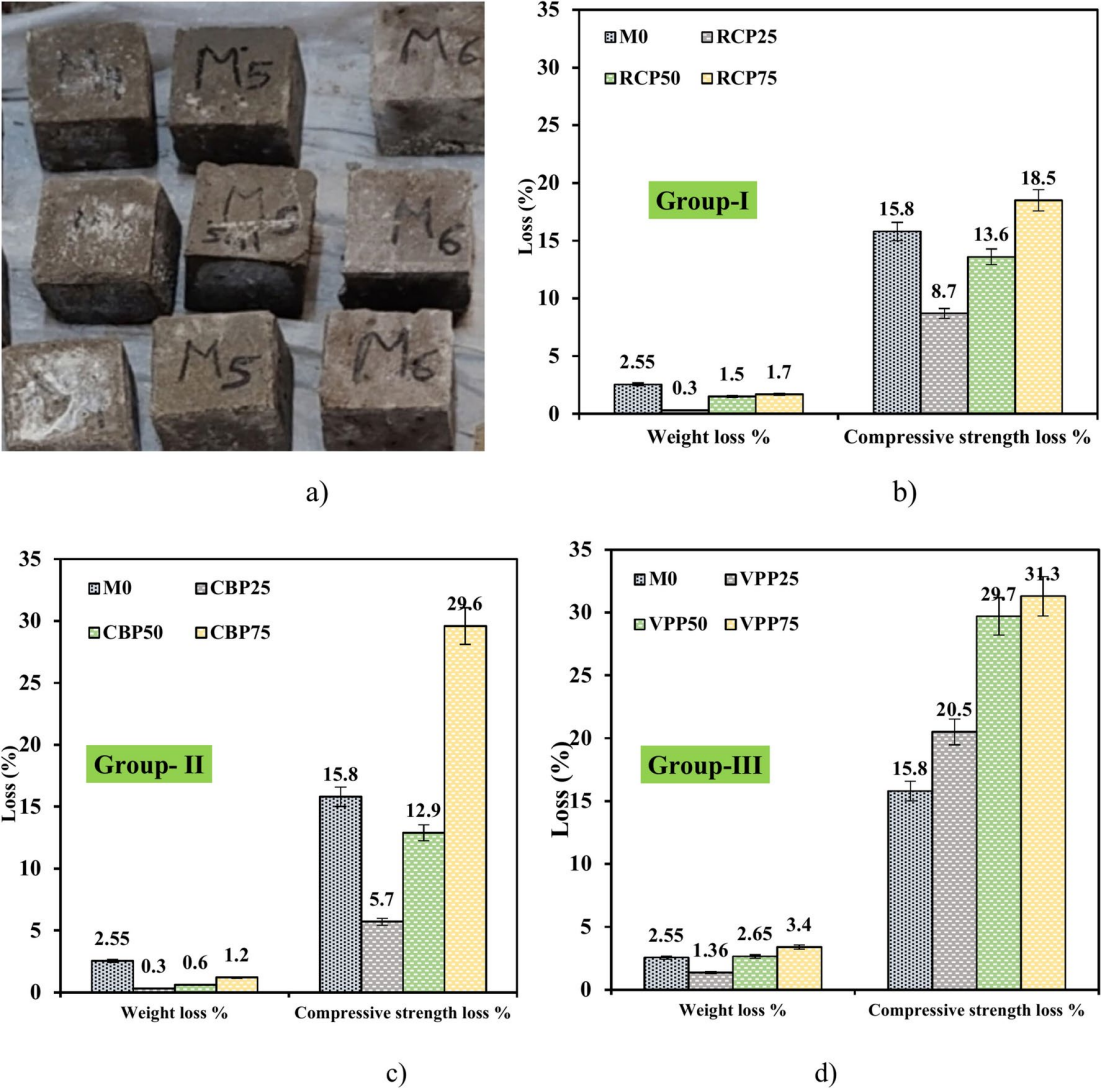
Impact of MgSO_4_ on weight and CS of prepared samples at 28 days.

Figure 11 presents the weight loss and CS reduction results after a 28-day immersion period in a 5% MgSO₄ solution. All mixtures successfully passed sulfate resistance testing, meeting the requirements of ASTM C-267. The study demonstrated significant differences in weight and strength change behaviors among the mixtures, reflecting the impact of replacement materials on the geopolymer matrix’s durability. For the M0 mixture, a weight decreases of 2.55% was observed, attributed to the formation of gypsum and ettringite as secondary products of sulfate exposure. These compounds temporarily fill voids, leading to mass gain, as previously reported by^12,36^ (see Fig. 11a and b). However, the CS of M0 decreased by 15.8%, indicating that structural integrity was compromised due to internal microstructural changes.

In mixtures incorporating RCP, the weight initially decreased by 0.3% in the RCP25 sample, likely due to RCP’s residual cementitious properties enhancing sulfate resistance. However, higher RCP replacement levels (RCP50 and RCP75) resulted in weight reductions of 1.5% and 1.7%, respectively. CS loss followed a similar trend, with reductions of 8.7%, 13.6%, and 1.5% for RCP25, RCP50, and RCP75, respectively^37–39^. Similarly, mixtures with CBP showed minimal weight reductions of 1.2%, 0.6%, and 0.3% for CBP25, CBP50, and CBP75, respectively (Fig. 11c). CS loss for these mixtures was relatively low, recorded at 5.7%, 12.9%, and 29.6%, respectively. Conversely, mixtures containing VPP exhibited higher weight reductions of 1.36%, 2.65%, and 3.4% for VPP25, VPP50, and VPP75, respectively, with corresponding CS reductions of 20.5%, 29.7%, and 31.3% (Fig. 11d). This behavior may be attributed to VPP’s limited pozzolanic activity at higher replacement levels, which compromises matrix integrity over time. These findings reveal the essential role of material selection, as factors like chemical composition, particle structure, and interaction with alkaline activators significantly influence the durability performance of GPC under sulfate exposure.

#### Acid attack: weight and strength changes

**Fig. 12:**
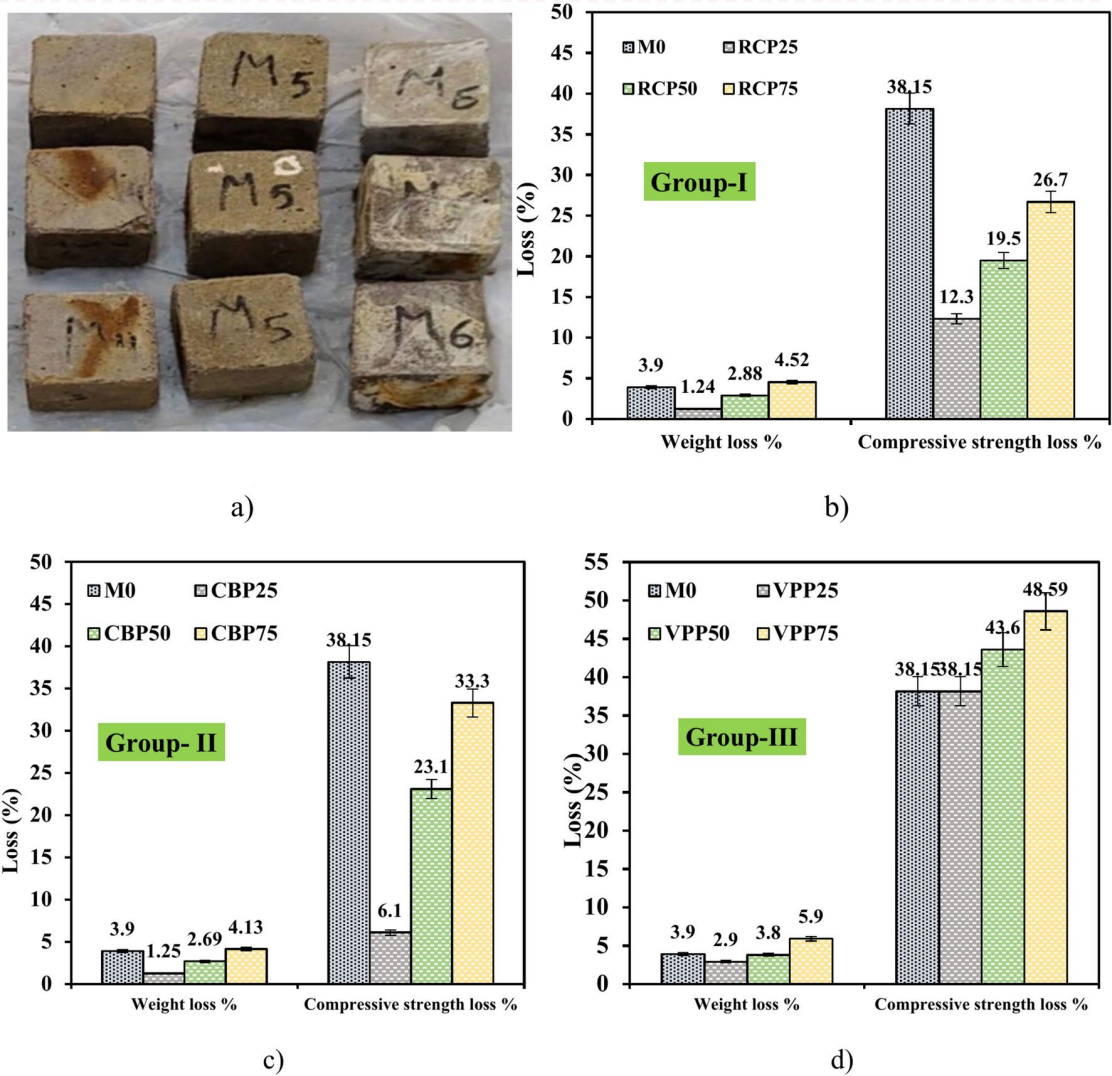
Impact of H_2_SO_4_ on weight and CS of prepared samples at 28 days.

Figure 12 presents the weight loss and CS reduction results after a 28-day immersion period in a 5% H₂SO₄ solution. For the M0 mixture, a weight decreases of 3.9% was observed, attributed to the formation of gypsum and ettringite as secondary products of acid exposure. However, the CS of M0 decreased by 39.15%, indicating structural integrity was significantly compromised due to acid-induced leaching and internal microstructural degradation. In mixtures incorporating RCP (Fig. 12b), the weight initially decreased by 1.24% in the RCP25 sample, likely due to RCP’s residual cementitious properties enhancing acid resistance. Higher RCP replacement levels (RCP50 and RCP75) resulted in weight reductions of 2.88% and 4.55%, respectively. CS loss followed a similar trend, with reductions of 12.3%, 19.5%, and 26.7% for RCP25, RCP50, and RCP75, respectively. These results align with findings by^34,37,39^, which suggest that excessive RCP can introduce microcracks and increase porosity, leading to both weight loss and strength degradation under acidic conditions. Similarly, mixtures with CBP exhibited minimal weight reductions of 1.25%, 2.69%, and 4.13% for CBP25, CBP50, and CBP75, respectively (Fig. 12c). CS loss for these mixtures was relatively low, recorded at 6.1%, 23.1%, and 33.3%, respectively. These results demonstrate CBP’s positive contribution to both weight and strength retention, likely due to its stable particle structure and reduced reactivity with acidic solutions, which protects the geopolymer matrix. Conversely, mixtures containing VPP exhibited higher weight reductions of 2.9%, 3.8%, and 5.9% for VPP25, VPP50, and VPP75, respectively (Fig. 12d). CS reductions were similarly significant, measured at 38.15%, 43.9%, and 48.59%. When compared to other waste materials, CBP exhibited the most favorable performance in both weight and CS retention, followed by RCP, with VPP being the least effective. While RCP offers moderate acid resistance and contributes to matrix densification at lower replacement levels, excessive incorporation diminishes durability, as noted in^23,26,34,37,39^.

#### Residual compressive strength and mass loss

**Fig. 13:**
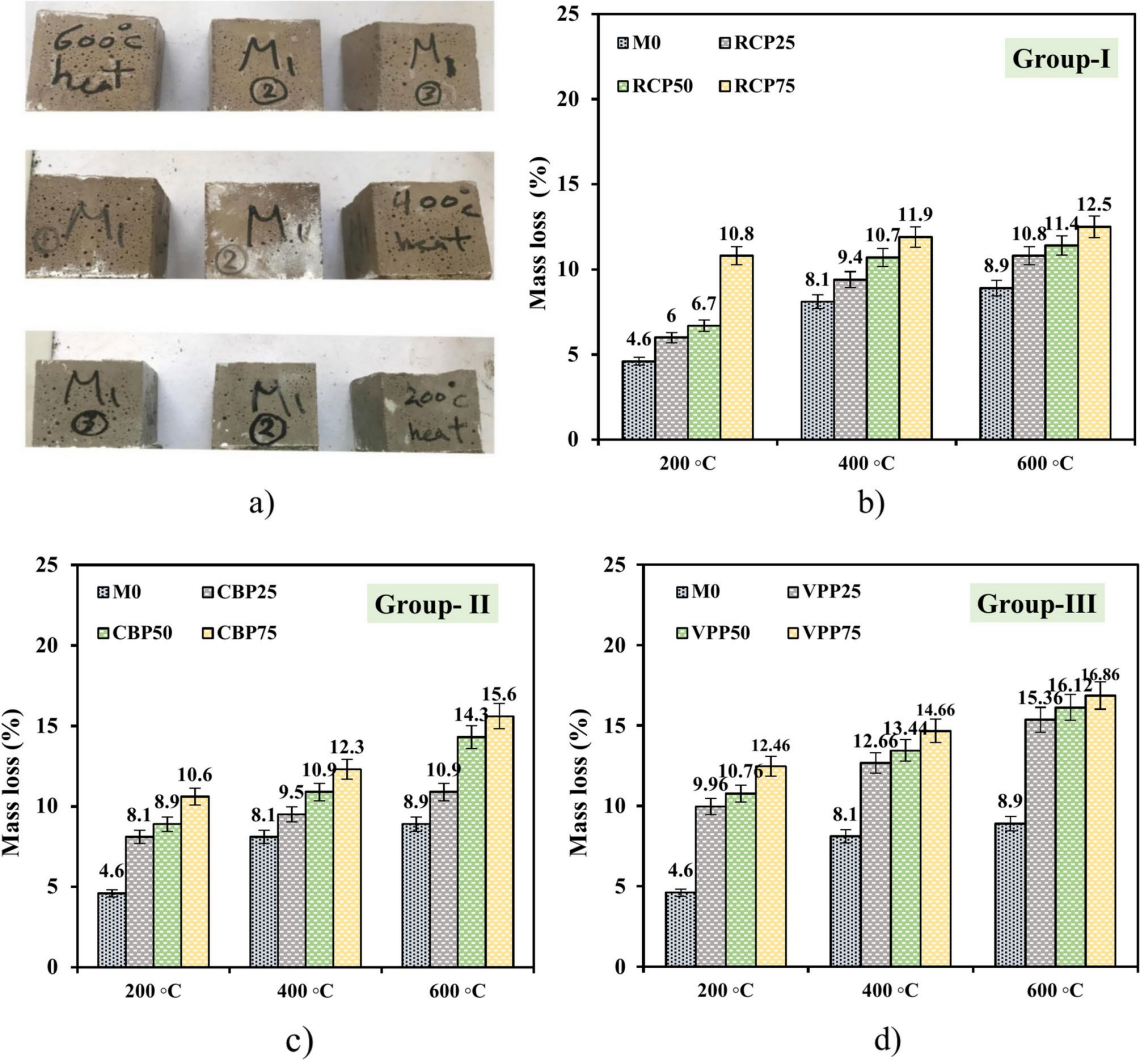
Mass loss of GPC samples after exposure to elevated temperatures.

**Fig. 14:**
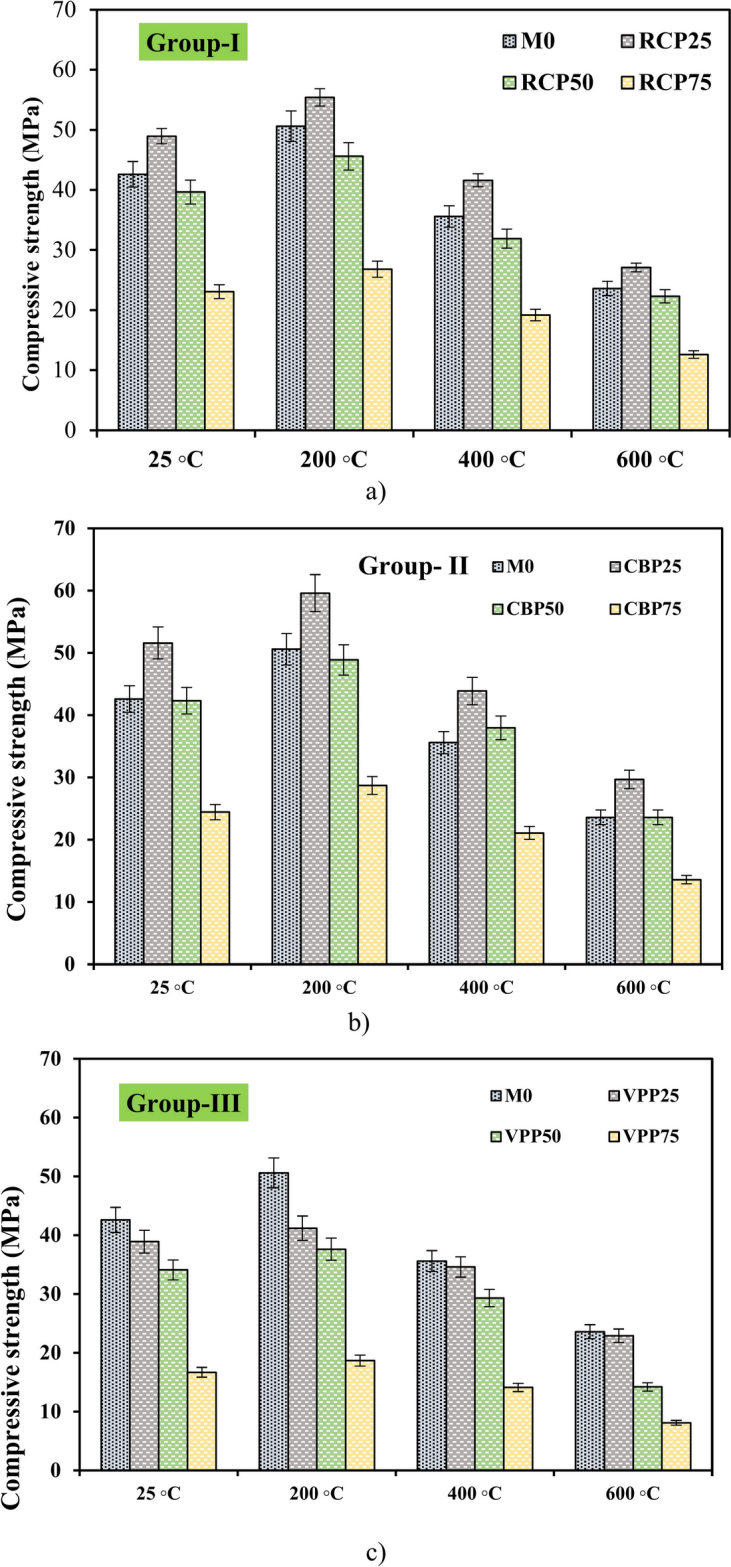
Residual compressive strength of GPC samples at elevated temperature.

Figure 13 illustrates the mass loss and residual CS of the prepared mixtures after exposure to elevated temperatures. The impact of high temperatures on GPC varied depending on the type of supplementary cementitious material incorporated. Among the prepared mixtures, VPP25 exhibited the highest mass loss rates, with values of 12.46%, 16.12%, and 16.8% at 200 °C, 400 °C, and 600 °C, respectively (Fig. 13). At 600 °C, the mass loss for RCP25 and CBP25 samples was 12.5% and 15.6%, respectively. Notably, RCP25 demonstrated the lowest mass loss, indicating superior resistance to high temperatures. This can be related to the optimal proportion of RCP particles, which provide pozzolanic activity and a micro-filling effect, enhancing the material’s thermal stability. In comparison to previous studies^1,20,22,40^, the mass loss of GPC at 600 °C was more significant. Research by Tahwia et al.^41^ reported that GPC incorporating waste materials exhibited mass losses ranging from 8 to 10% at similar temperatures. However, these systems lacked the inclusion of recycled materials, which could contribute to higher thermal decomposition. Similarly, a study by Topal et al.^42^ noted that traditional geopolymer systems showed better retention of mass due to their higher silica content, which contributed to enhanced structural stability under thermal stress. Compared to previous research^1,20,22,40^, GPC exhibited more pronounced mass loss at 600 °C, primarily due to the decomposition of mineral components in both the paste and aggregates, regardless of the waste material content. The compressive strength of GPC also decreased significantly at 600 °C, as shown in Fig. 14. The observed weight loss and strength reduction were primarily caused by the evaporation of free water and the dehydration of partially hydrated water.

The RCP25 mixture retained a residual CS of 27.1 MPa (Fig. 14a), maintaining 44.62% of its original strength, whereas the M0 sample retained 23.6 MPa after exposure to 600 °C. At the same temperature, the CBP25 and VPP25 samples exhibited CS losses of 42.44% and 58.35%, respectively, compared to their strengths under water curing (Fig. 14b and c). These findings align with those of Neville et al.^43^, who demonstrated that concrete incorporating pozzolanic materials generally performs better at elevated temperatures due to reduced thermal expansion and improved microstructural integrity. Notably, the internal thermal stresses increased with rising temperatures, leading to more significant structural damage and reduced strength. This observation agrees with the study by Khan et al.^44^, which reported that concrete exposed to 600 °C experienced significant internal cracking due to differential thermal expansion between the paste and aggregate phases. However, the use of RCP in this study mitigated some of these effects by enhancing the paste-aggregate bond and providing micro-filling properties that reduced voids and thermal stress concentrations. Hence, while the thermal performance of GPC in this study is comparable to previous works, the use of waste materials such as RCP and VPP introduces variability in thermal stability and strength retention. This demonstrates the need for optimized material formulations to achieve superior thermal resistance in sustainable concrete systems^8,15,45^.

### Microscopic investigations

**Fig. 15:**
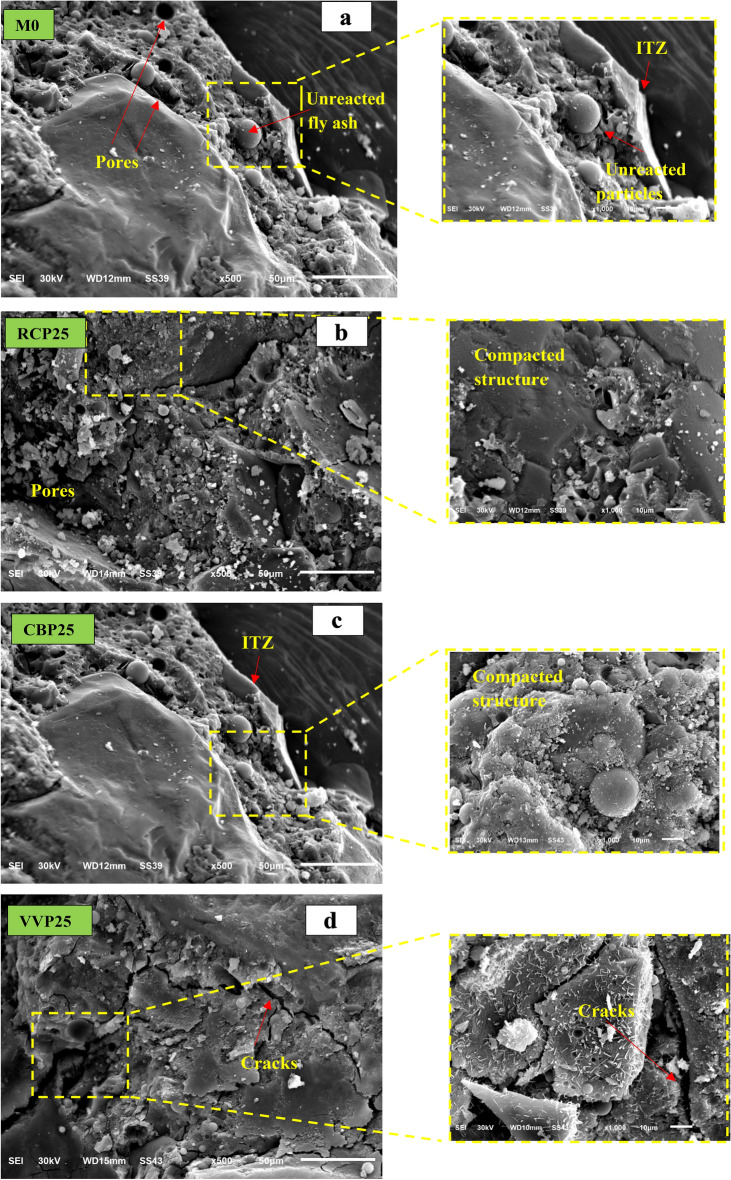
SEM morphologies of prepared GPC samples under water curing.

Microstructural investigations were conducted on GPC mixtures, including the M0 and optimized samples (RCP25, CBP25, and VPP25), to examine the relationship between microstructural variations and the observed mechanical and durability properties. It is well established that pore structure, reaction products, and the ITZ significantly impact on the microstructure and performance of GPC^37,46^. Unlike PC concrete, GPC develops its structure through alkali activation, which produces a three-dimensional aluminosilicate gel network, commonly referred to as N–A–S–H gel, alongside varying quantities of C–A–S–H or C–S–H gels depending on the raw materials used. In the M0 sample, SEM images revealed a porous microstructure with visible microcracks and unreacted particles (Fig. 15a). The ITZ appeared as a weak, poorly bonded region with high porosity, negatively impacting mechanical performance and durability. Additionally, voids and unevenly distributed geopolymer gel phases were observed, consistent with previous studies indicating that improper alkali-to-binder ratios can hinder the densification of the geopolymer matrix^37,46^. For RCP25 and CBP25 mixtures, SEM micrographs (Fig. 15b and c) showed a denser and more compacted microstructure compared to M0 and VPP25. The reduced porosity and refined pore structure correlate with the observed improvements in mechanical properties and durability performance. The pozzolanic reaction of RCP and CBP, driven by their high silica content, enhances the formation of N–A–S–H gel and results in a denser matrix. Interestingly, while VPP25 also demonstrated a reduction in pore size (Fig. 15d), its microstructure was less compacted than that of RCP25 and CBP25. SEM analysis revealed more microcracks and unreacted volcanic particles, contributing to its relatively lower mechanical performance.

**Fig. 16:**
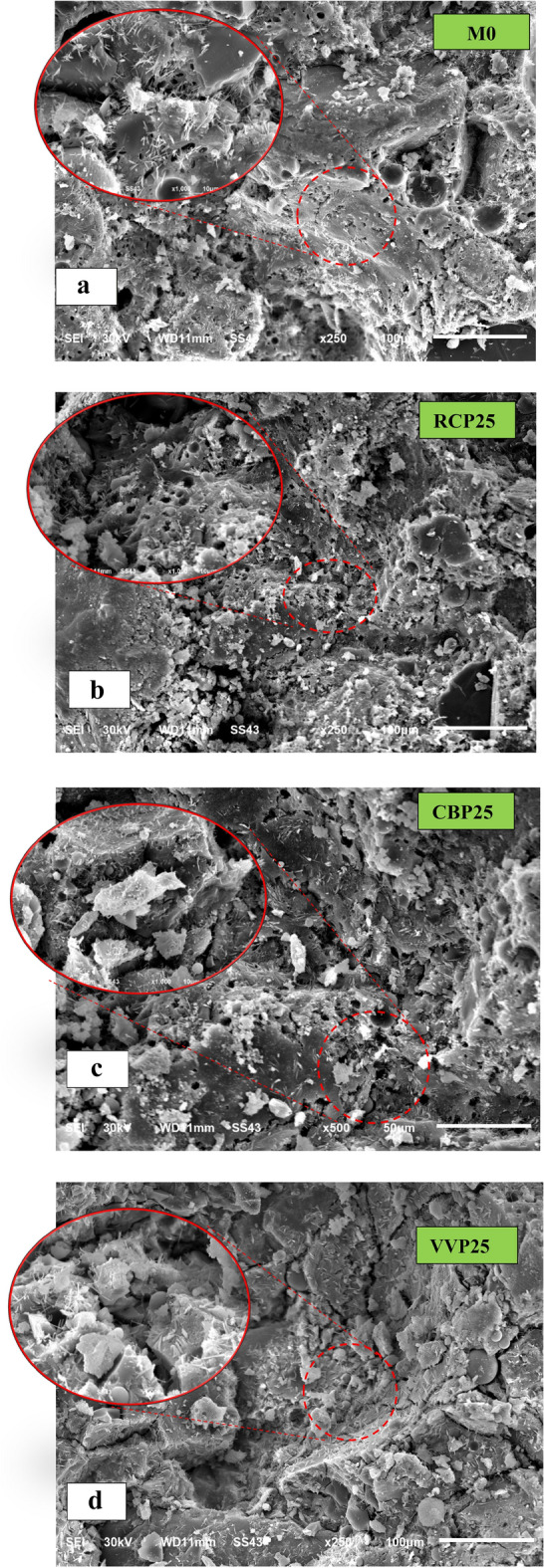
SEM images of prepared GPC samples after exposed to 400 °C.

At elevated temperatures (400 °C), significant structural degradation was observed in all GPC samples (Fig. 16). The control mixture (M0) exhibited large cracks, indicating extensive thermal damage. SEM images (Fig. 16a) showed the ITZ had almost completely deteriorated, with visible delamination and separation between geopolymer gel phases. The transformation of N–A–S–H gel into disconnected and dispersed phases under thermal stress led to a significant reduction in mechanical strength. In contrast, RCP25, CBP25, and VPP25 mixtures exhibited superior thermal resistance, as evidenced by their relatively intact internal microstructures. Despite some degree of thermal damage, the ITZ in these mixtures remained more cohesive, and the geopolymer gels were tightly interconnected. For RCP25 and CBP25, this behavior can be attributed to their enhanced pozzolanic reactivity, which promotes the formation of additional N–A–S–H gels under thermal conditions. This observation aligns with findings by^47^, who demonstrated that pozzolanic additives enhance thermal stability in GPC by forming thermally resistant aluminosilicate networks. VPP25, while showing better thermal resistance than the control, exhibited more pronounced microcracks compared to RCP25 and CBP25 (Fig. 16b and c). This is likely due to the lower reactivity of volcanic particles, which limits the formation of a dense geopolymer matrix capable of withstanding high temperatures. Similar conclusions were drawn by^35,37,46^, who noted that low-reactivity precursors result in weaker thermal resistance in GPC. The observed microstructural differences provide a clear explanation for the variations in mechanical and durability performance among the mixtures. The compacted microstructures of RCP25 and CBP25 reduce porosity, mitigate crack propagation, and improve load transfer across the matrix, resulting in superior strength and durability under both ambient and elevated temperature conditions. Meanwhile, the less dense and cracked microstructure of VPP25 correlates with its lower performance metrics Fig. 16d. These findings confirm that the addition of pozzolanic materials like RCP and CBP not only improves microstructural compactness but also enhances thermal stability by generating additional geopolymer gel phases.

**Fig. 17:**
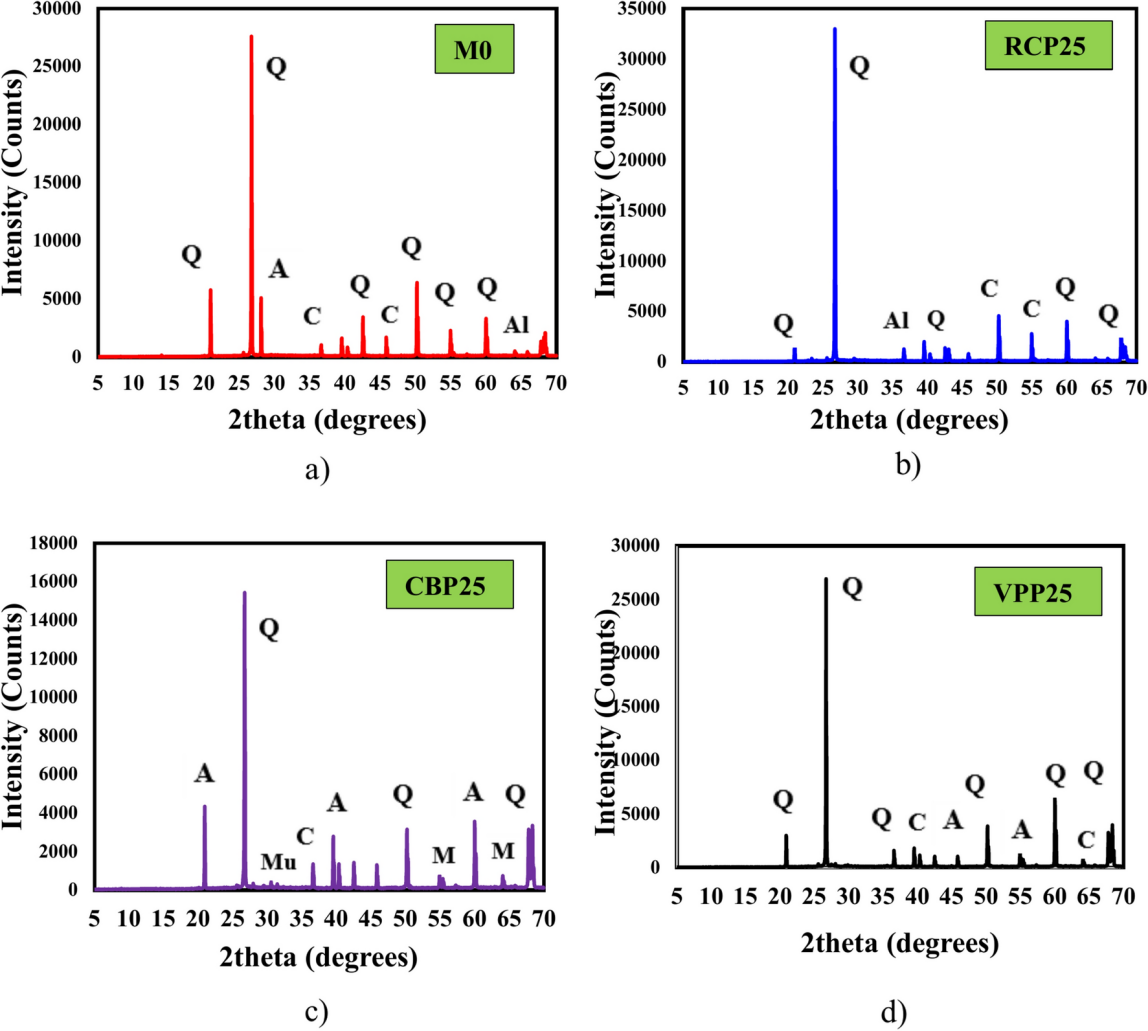
XRD analysis of prepared GPC (SiO_2_ (quartz—Q)—CaAl_2_Si_2_O_8_ (anorthite—A)—Al_6_Si_2_O1_3_ (mullite—Mu)—NaAlSi_3_O_8_ (allbite—Al)—Muscovite (KF)₂(Al₂O₃)₃(SiO₂)₆−M−CaCO_3_ (calcite- C).

In addition, the comparative analysis of XRD patterns reveals the mineralogical composition and crystalline phases in the GPC mixtures (control mixture, RCP25, CBP25, and VPP25), as shown in Fig. 17. Sun and Vollpracht reported that, due to geopolymerization, a shift of the amorphous hump to higher 2θ angles can be observed in Fig. 17, indicating the formation of a new amorphous phase^33,48^. This new amorphous phase is characterized as a highly cross-linked, structurally disordered aluminosilicate gel, commonly represented as N–A–S–(H)^1^. For the control GPC mixture (M0), a prominent peak appears around the 20–40° diffraction angle, indicating the formation of polymerization products, specifically C–(N)–A–S–H gels, as reported by previous studies^19,33,48^. In the RCP25 mixture (Fig. 17b), XRD patterns display dominant peaks corresponding to quartz, calcite, and minor traces of albite. The presence of calcite is associated with carbonation in RCP^49^. Additionally, broad humps around 20–35° 2θ suggest an amorphous geopolymer gel network containing C–S–H and N–A–S–H phases, contributing to strength development and matrix densification^19^. For the CBP25 mixture (Fig. 17c), prominent quartz peaks, along with muscovite and anorthite, are observed, reflecting the mineralogical characteristics of clay brick powder. Compared to RCP25, CBP25 exhibits a more pronounced amorphous hump, indicating higher reactivity, which promotes extensive geopolymerization and results in a dense, interconnected N–A–S–H gel network^33,48^. In contrast, the VPP25 mixture shows crystalline phases like quartz and feldspar (anorthite), consistent with the mineralogy of volcanic pumice (Fig. 17d). However, the less prominent amorphous hump reflects lower reactivity. This is accompanied by weaker C–S–H peaks and a less compacted N–A–S–H network, leading to a more porous microstructure and lower mechanical performance. This decline is attributed to the incorporation of these pozzolanic additives, which influences the availability of dissolved Al and Si in the geopolymer matrix^28,34,50^. Consequently, the reduced dissolution of aluminosilicate precursors limits the development of a robust three-dimensional amorphous aluminosilicate network, affecting the overall geopolymerization process and impacting the material’s mechanical and durability properties. Therefore, these findings highlight the significant influence of precursor type on gel phase development in GPC, with CBP25 demonstrating superior performance, RCP25 offering balanced properties, and VPP25 being constrained by lower reactivity.

## Conclusions

The aim of this study is to investigate the effects of incorporating RCP, CBP, and VPP as partial replacements for fly ash and GGBS in GPC. The research focuses on evaluating the mechanical properties, durability, and thermal performance of the developed geopolymer mixtures under water curing. The key findings of the research are summarized as follows:

1. RCP exhibited a higher water demand (97.96 kg/m^3^) due to its fine particle size and high surface area, CBP demonstrated the highest water demand (111.71 kg/m^3^) attributed to its fine, porous nature and the presence of reactive silica and alumina, while VPP required the least water (65.34 kg/m^3^) due to its lower specific surface area and porous morphology, which enhanced mix fluidity.
2. Low replacement levels (25%) of RCP and CBP enhanced CS due to the filler effect, matrix densification, and pozzolanic reactivity. RCP25 improved CS by 14.88%, while CBP25 achieved the highest increase at 21.12% after 28 days. Conversely, VPP25 reduced CS by 8.68% due to its porous structure, which introduced voids and weakened the matrix. At higher replacement levels (50–75%), all mixtures exhibited reduced CS due to increased porosity, binder dilution, and weaker bonding caused by microcracks and unreactive impurities.
3. M0 demonstrated the highest sulfate resistance, with minimal weight loss (0.42%) and CS reduction (3.5%). Among the waste-based replacements, CBP25 showed superior resistance with a weight loss of 0.48% and CS reduction of 4.1%, outperforming RCP25, which exhibited a weight loss of 0.65% and a CS reduction of 5.8%. VPP25 experienced the most significant degradation, with a weight loss of 1.03% (145% higher than M0) and a CS reduction of 9.7%, indicating its reduced resistance to sulfate attack.
4. Acid exposure resulted in weight losses of 1.6% for M0, 2.2% for RCP25, 1.8% for CBP25, and 4.5% for VPP25. CBP25 demonstrated the lowest weight loss among waste-based replacements, indicating superior acid resistance. Compressive strength reductions followed a similar trend, with M0 showing the least reduction (6.8%), followed by CBP25 (7.1%), RCP25 (8.9%), and VPP25 (13.2%). VPP25 exhibited the highest degradation under acid attack, with a weight loss 181% greater and a CS reduction nearly double that of M0.
5. The thermal resistance at 600 °C showed an increase in mass loss from 9.4% for M0 to 10.7% for RCP25, 11.1% for CBP25, and 12.9% for VPP25. In terms of compressive strength retention, M0 retained the highest strength at 47.2%, followed by RCP25 at 44.62%, CBP25 at 41.8%, and VPP25 at 38.5%. RCP25 demonstrated moderate thermal performance, retaining more strength despite higher mass loss compared to other modified binders.

## Data Availability

The data that support the findings of this study are available from the corresponding author, [Mohamed Abdellatief: best time703@std.mans.edu.eg], upon reasonable request.
